# Application of Machine Learning Prediction of Individual SARS-CoV-2 Vaccination and Infection Status to the French Serosurveillance Survey From March 2020 to 2022: Cross-Sectional Study

**DOI:** 10.2196/46898

**Published:** 2023-11-28

**Authors:** Stéphanie Bougeard, Adeline Huneau-Salaun, Mikael Attia, Jean-Baptiste Richard, Caroline Demeret, Johnny Platon, Virginie Allain, Stéphane Le Vu, Sophie Goyard, Véronique Gillon, Sibylle Bernard-Stoecklin, Bernadette Crescenzo-Chaigne, Gabrielle Jones, Nicolas Rose, Sylvie van der Werf, Olivier Lantz, Thierry Rose, Harold Noël

**Affiliations:** 1 Epidemiology, Health and Welfare Laboratory of Ploufragan-Plouzané-Niort French Agency for Food, Environmental, Occupational Health & Safety Ploufragan France; 2 Unit of Molecular Genetics of RNA Viruses Institut Pasteur Paris France; 3 Data Support, Processing and Analysis Department Santé publique France Saint-Maurice France; 4 EPI-PHARE Saint-Denis France; 5 Diagnostic Test Innovation and Development Core Facility Institut Pasteur Paris France; 6 Clinical Immunology Laboratory Institut Curie Paris France; 7 Infectious Disease Division Santé publique France Saint-Maurice France

**Keywords:** SARS-CoV-2, serological surveillance, infection, vaccination, machine learning, seroprevalence, blood testing, immunity, survey, vaccine response, French population, prediction

## Abstract

**Background:**

The seroprevalence of SARS-CoV-2 infection in the French population was estimated with a representative, repeated cross-sectional survey based on residual sera from routine blood testing. These data contained no information on infection or vaccination status, thus limiting the ability to detail changes observed in the immunity level of the population over time.

**Objective:**

Our aim is to predict the infected or vaccinated status of individuals in the French serosurveillance survey based only on the results of serological assays. Reference data on longitudinal serological profiles of seronegative, infected, and vaccinated individuals from another French cohort were used to build the predictive model.

**Methods:**

A model of individual vaccination or infection status with respect to SARS-CoV-2 obtained from a machine learning procedure was proposed based on 3 complementary serological assays. This model was applied to the French nationwide serosurveillance survey from March 2020 to March 2022 to estimate the proportions of the population that were negative, infected, vaccinated, or infected and vaccinated.

**Results:**

From February 2021 to March 2022, the estimated percentage of infected and unvaccinated individuals in France increased from 7.5% to 16.8%. During this period, the estimated percentage increased from 3.6% to 45.2% for vaccinated and uninfected individuals and from 2.1% to 29.1% for vaccinated and infected individuals. The decrease in the seronegative population can be largely attributed to vaccination.

**Conclusions:**

Combining results from the serosurveillance survey with more complete data from another longitudinal cohort completes the information retrieved from serosurveillance while keeping its protocol simple and easy to implement.

## Introduction

In the ongoing global effort to contain the SARS-CoV-2 pandemic, population-wide serological surveys are recommended for disease surveillance and policymaking [1]. Serological assays directly measure the antibody response to SARS-CoV-2 resulting from viral infection or vaccination. Monitoring seroprevalence is of paramount interest to complement case-based surveillance that does not capture subclinical cases or people using self-tests and to evaluate the effectiveness of the vaccination strategy over time. Therefore, the World Health Organization’s UNITY initiative promotes serological surveys by providing guidelines to standardize worldwide serological studies.

Despite the availability of well-known methods as well as guidelines, implementation of serological surveys remains challenging in terms of the resources and logistic means needed to obtain samples [2]. Collecting residual sera from routine clinical blood testing represents an easy and inexpensive solution. This strategy was chosen for the nationwide SARS-CoV-2 serosurvey in France (SERPICO) [3]. This survey, conducted by the national health agency Santé publique France and the National Reference Centre for Respiratory Infections Viruses of the Institut Pasteur, aimed to estimate the seroprevalence of anti–SARS-CoV-2 antibodies in the French population over time according to gender, age, and region. The humoral immune status of individuals was determined by 3 complementary serological assays: 2 luciferase-linked immunosorbent assays (LuLISAs) detecting the nucleocapsid (LN) and the spike (LS) proteins of SARS-CoV-2 and a pseudoneutralization assay (PNT). In total, 8 collection periods between March 2020 and March 2022 monitored the evolution of seroprevalence in the French general population.

Substantial differences were reported in the effectiveness and duration of natural versus vaccine-conferred or hybrid immunity against SARS-CoV-2 reinfection [4,5]. In particular, prior infection after 1 dose of vaccine elicited antispike IgG antibody responses with higher peak levels or longer half-lives than 1 or 2 vaccinations in seronegative individuals [6]. We therefore aimed to reconstruct the proportion of infected versus vaccinated or infected and vaccinated individuals over time as it may carry meaningful lessons and potential applications for future vaccine-preventable disease pandemics.

Although the SERPICO serosurvey lacked data documenting the status of the sampled individuals with respect to previous SARS-CoV-2 infection or vaccination, the detailed serological data presented here could allow novel approaches to monitor the SARS-CoV-2 seroprevalence of the French population with regard to infection or vaccination.

In this study, we propose a model derived from a machine learning procedure to predict individuals’ immune status with respect to SARS-CoV-2 infection and vaccination based on results from 3 serological assays. We applied this predictive model to the SERPICO serosurvey to characterize immune status resulting from natural infection and vaccination for the French population between 2020 and 2022.

## Methods

### Serological Assays

The National Reference Centre for Respiratory Infections Viruses and the Diagnostic Test Innovation and Development core facility of the Institut Pasteur developed 3 serological assays: 2 LuLISAs detecting the LN and LS proteins of SARS-CoV-2 and a PNT [7]. These serological results are considered explanatory variables in the predictive models. They were expressed as log_10_ of their original value.

### Data

This study used 3 data sets (Table 1). The CURIE-O-SA [7] and prepandemic [3,7] data sets were used to build and validate the model, and the SERPICO [3] data set was used for application of the model.

The CURIE-O-SA study is a large cohort of 1917 workers in a hospital and research center specialized in oncology. Of the 4394 individuals included in this data set, 77% (n=3595) were men and 23% (n=899) were women. The median age was 38 (IQR 19-82) years with 94% (n=4130) of participants between 20 and 59 years of age. The CURIE-O-SA serological assay results were available at different sampling times (“individual date”) with a mean number of times per participant equal to 2.5 (SD 1.2) and 73% (n=3208) of individuals with 1, 2, or 3 sampling times. Information on SARS-CoV-2 history (symptoms and date of positive reverse transcription polymerase chain reaction [RT-PCR] test, if any) and on SARS-CoV-2 vaccination (number of injections and dates) was collected. The uninfected status (0) was set if the following 3 conditions were met: no positive PCR test, no declaration of ageusia or anosmia, and a log_10_LN value below 4.60. Individuals with log_10_LN>4.60 and no positive PCR test were considered potential subclinical cases and were excluded. The infected status (1) was set for individuals with a positive RT-PCR result history only; individuals reporting symptoms for SARS-CoV-2 with no positive RT-PCR result were excluded. The vaccination status (0=unvaccinated, 1=1 injection, 2=2 injections) was defined while taking into account a delay of 15 days after vaccination (immunity onset) and no more than 6 months after the last injection (immunity waning). Of the 4394 results, 80.4% (n=3532) of results were negative (uninfected and unvaccinated), 5.2% (n=231) were infected and unvaccinated, 10.9% (n=477) were vaccinated twice, 1.9% (n=82) were vaccinated once, and 1.6% (n=72) were infected and vaccinated.

The prepandemic results came from healthy donors from a blood bank before 2019 and were expected to be predicted negative (uninfected and unvaccinated) by the model. In this data set, of the 233 individuals, 32.2% (n=75) were men and 67.8% (n=158) were women with a median age of 44 (IQR 18-81) years.

The SERPICO data were considered application data for the model. The SERPICO survey monitored the evolution of seroprevalence of anti–SARS-CoV-2 antibodies in the French population (mainland area) from March 2020 to March 2022 with 8 periods of sampling. Results for the 3 serological assays (LN, LS, and PNT) were available for 23,886 samples.

**Table 1:** Description of the 3 data sets under study.

Data set	Description	Use	Number of results	Variables
CURIE-O-SA [7] (n=4394)	Cohort study among health workers; April 2020-November 2021	Reference for SARS-CoV-2 status; reference for SARS-CoV-2 vaccination status	4394 individuals × dates	LS^a^, LN^b^, PNT^c^ values; gender; age; SARS-CoV-2 infection status (0=uninfected, 1= infected); SARS-CoV-2 vaccination status (0=unvaccinated, 1=1 injection, 2=2 injections)
Prepandemic [3,7] (n=233)	Blood donors; 2014-2018	Control for uninfected SARS-CoV-2 status; control for unvaccinated SARS-CoV-2 status	233 individuals	LS, LN, PNT values; gender; age
SERPICO [3] (n=23,886)	Transversal nationwide survey, residual sera; March 2020-March 2022	Application	23,886 individuals × dates	LS, LN, PNT values; gender; age; region

^a^LN: nucleocapsid.

^b^LS: spike protein.

^c^PNT: pseudoneutralization assay.

### Machine Learning Procedure

**Figure 1:**
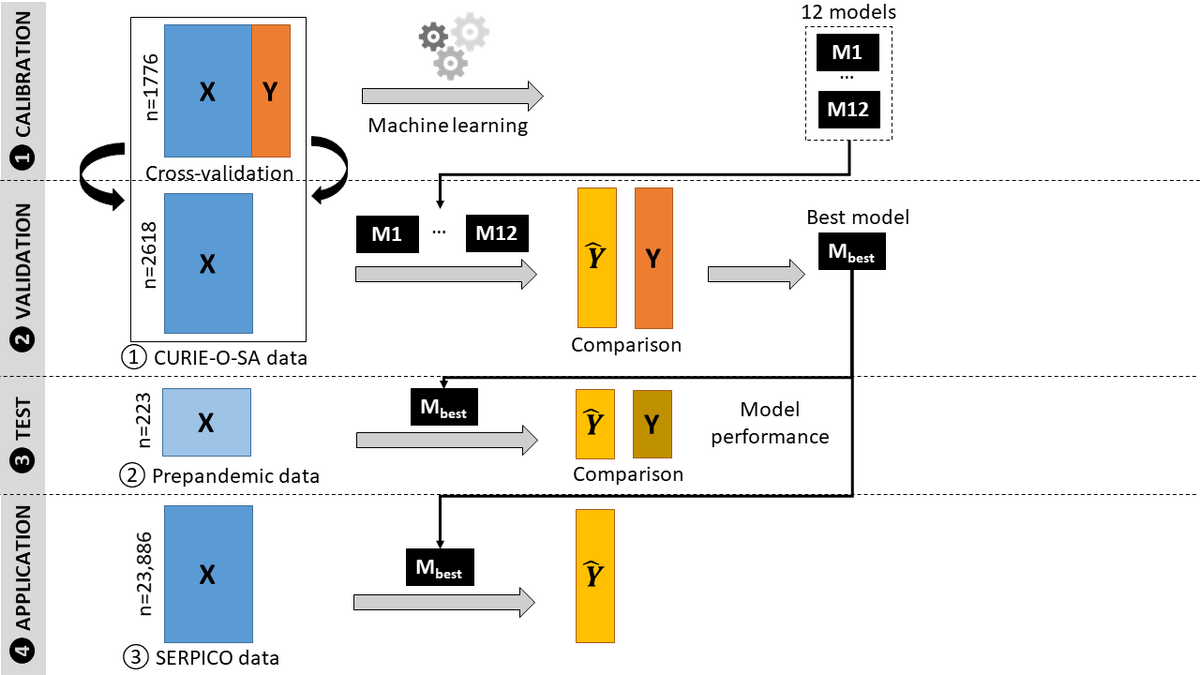
Sketch graph of the 3-step model-building procedure (calibration, validation, and testing) and the application step. Regardless of the data set (ie, CURIE-O-SA, prepandemic, or SERPICO), the X explanatory data contained the log_10_ values of the 3 serological assays (log_10_LN, log_10_LS, and log_10_PNT) and the Y outcome data, with the SARS-CoV-2 status being known (CURIE-O-SA, prepandemic) or unknown (SERPICO).

The model was built using a 4-step procedure involving calibration, validation, testing, and application (Figure 1). It aimed to predict the SARS-CoV-2 status at the individual level from 3 serological assays (LS, LN, and PNT) and 2 covariables (age and gender). The SARS-CoV-2 status was defined as a variable with 5 modalities: uninfected-unvaccinated (NEG), vaccinated with 1 dose (VAC1), vaccinated with 2 doses (VAC2), infected-unvaccinated (INF), and infected-vaccinated regardless of the number of doses (INF.VAC). A machine learning procedure was applied to challenge several classification models [8]. The model with the best predictive performance was selected from a cross-validation procedure on the first data set (CURIE-O-SA) then validated on an external data set (prepandemic). Finally, the selected model was applied to the French nationwide serosurveillance survey (SERPICO) to infer the proportions of the 5 SARS-CoV-2 statuses.

More precisely, 12 parametric and nonparametric classification models from 5 main statistical families were assessed within a machine-learning procedure. The models came from the following families: (1) generalized linear models (standard multinomial regression, penalized generalized linear model), (2) Bayesian models (naive Bayesian classification), (3) factor analysis models (discriminant analysis, mixture discriminant analysis, regularized discriminant analysis, kernel partial least squares discriminant analysis), (4) decision tree models (bagged classification and regression tree, Quinlan’s C5.0 algorithm), and (5) nonparametric classification models (K nearest neighbors, support vector machine, neural network) [9]. To select the best predictive model and avoid overfitting, a repeated (200 times) 2-fold cross-validation was applied. To ensure the independence of the “individual x date” units, calibration data were randomly selected so that there was only 1 sample per individual involved in the CURIE-O-SA cohort; the remaining data were used for validation. The model parameters were optimized by means of a 10-fold cross-validation procedure on the calibration data. Percentages of well-classified samples for each modality of the status were calculated on the validation data. The best model was the one that best predicted the status modalities on average. Using the mean predictive performance of the 5 statuses to select the best model (rather than the overall prediction) can be considered oversampling of the rare modalities [10]. Repeated bootstrap simulations (50 times) were used to obtain 95% CIs. The overall procedure was implemented in R software (version 4.1.2, R Foundation for Statistical Computing) [11] by means of the “caret” package (version 6.0-90) [12].

### Ethical Considerations

The study was based on a secondary use of pseudonymized data collected from health professionals and already published [3,7]. According to French law, such studies are not required to receive ethics committee approval.

## Results

### Descriptive Statistics

**Figure 2:**
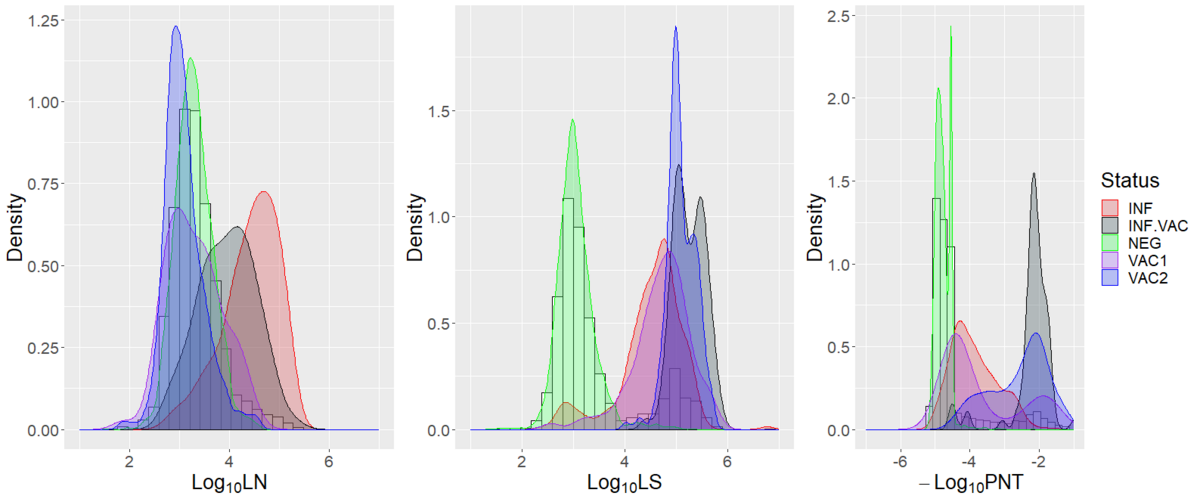
Density estimation of the log_10_ values of the serological assays (LN, LS, and PNT) according to their known SARS-CoV-2 status in the CURIE-O-SA cohort. INF: infected-unvaccinated; INF.VAC: infected-vaccinated regardless of the number of doses; LN: nucleocapsid; LS: spike protein; NEG: uninfected-unvaccinated; PNT: pseudoneutralization assay; VAC1: vaccinated with 1 dose; VAC2: vaccinated with 2 doses.

The CURIE-O-SA data set used to build and validate the predictive model is illustrated in Figure 2. The serological LN results differentiated INF and to a lesser extent INF.VAC individuals from the others. The serological results targeting the SARS-CoV-2 LS separated the NEG individuals from the others. INF individuals developed anti-LS and anti-LN protein immunoglobulins, while VAC1 and VAC2 individuals developed anti-LS immunoglobulins only. This difference in immunization between infected (and unvaccinated) and vaccinated individuals was expected in a cohort where VAC1 and VAC2 individuals were vaccinated by vaccines targeting the LS only. The PNT results separated the INF.VAC individuals from the others as INF.VAC individuals presented a higher response to the PNT assay than did VAC1 and VAC2 individuals.

The prepandemic data set used for model validation consisted of 223 negative sera with the following average values for the serological assays (log_10_ values): 3.16 (SD 0.30) for LN, 3.16 (SD 0.20) for LS, and 5.07 (SD 0.11) for PNT.

### Model

The percentages of well-classified results for each status and for all the models under study were calculated (Table 2).

**Figure 3:**
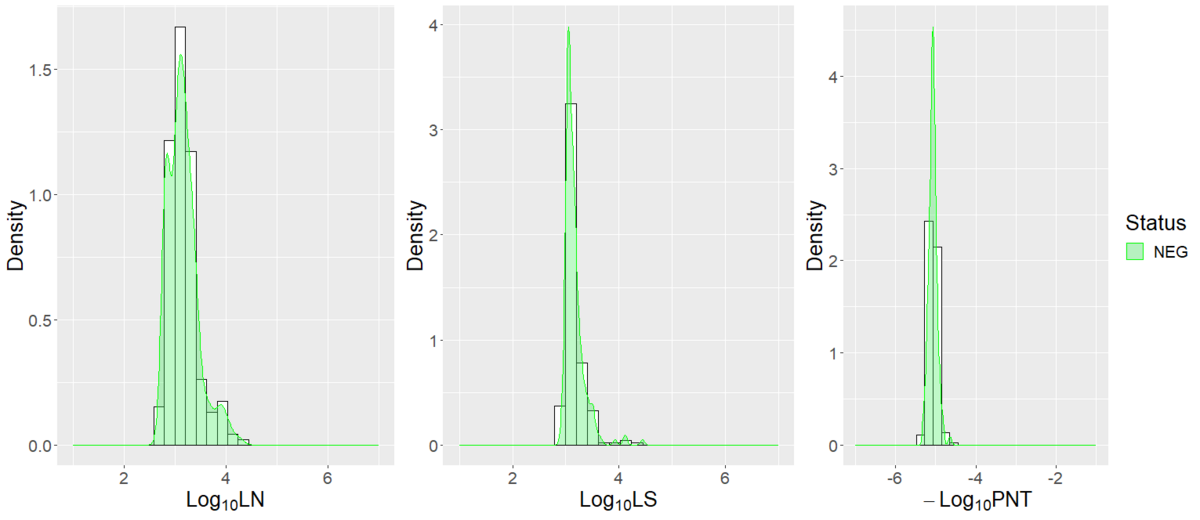
Density estimation of the log_10_ values of the serological assays (LN, LS, and PNT) according to their known SARS-CoV-2 status in the prepandemic data set. LN: nucleocapsid; LS: spike protein; NEG: uninfected-unvaccinated; PNT: pseudoneutralization assay.

The selected model was the mixture discriminant analysis with the best average prediction performance (mean 69.9%, SD 0.5%). More precisely, 98.4% (SD 0.2%) of the NEG status was well-predicted, as well as 40.6% (SD 6.9%) of the VAC1, 87.5% (SD 3.1%) of the VAC2, 42.6% (SD 11.3%) of the INF.VAC, and 78.9% (SD 3.8%) of the INF statuses. Best performances were obtained for the modalities with the largest numbers of “individual × date” units (NEG, VAC2, and INF). The VAC1 individuals were usually (60%) incorrectly predicted as VAC2; the error in the prediction did not depend on age or gender. The INF.VAC individuals were usually (84.4%) incorrectly predicted as VAC2; the error in the prediction did not depend on age and gender. As expected, this model predicted 99.5% of the negative prepandemic sera (Figure 3).

The model was applied to the prepandemic data (n=223). It predicted 222 results as negative and 1 as VAC1. This confirms the ability of the model to predict infection-free individuals, as 99.5% of the results were correctly predicted as NEG. This performance is in accordance with that calculated by means of cross-validation (98.4%). However, this performance is expected in that the model has many NEG values in the calibration data set (3532/4394; 80.4%) and is tested on expected NEG results. The only result not correctly predicted was predicted to be VAC1 with values log_10_LN=3.09, log_10_LS=4.44, and log_10_PNT=5.04. This individual had higher LS values than expected for NEG individuals (log_10_LN=3.28, log_10_LS=3.05, and log_10_PNT=4.78 for average NEG individuals in the CURIE-O-SA data set).

**Table 2:** Predictive performances of different models obtained by means of a repeated (200 times) 2-fold cross-validation using the CURIE-O-SA cohort (n=4394 individual × date units from April 2020-November 2021).

Model	Predictive performance (%), mean (SD)					
	NEG^a^ (n=3532)	VAC1^b^ (n=82)	VAC2^c^ (n=477)	INF.VAC^d^ (n=72)	INF^e^ (n=231)	Average^f^
Multinomial regression	99.1 (0.2)	3.4 (4.7)	95.4 (1.5)	29.1 (9.8)	68.1 (2.9)	59 (41.8)
Penalized generalized linear model	99.1 (0.2)	1.9 (3.6)	95.4 (1.3)	24.8 (12.6)	68 (2.8)	57.8 (43.1)
Naive Bayesian classification	98.5 (0.2)	6.7 (4.7)	91.3 (1.9)	47.8 (8.7)	83.8 (1.8)	65.6 (38.3)
Linear discriminant analysis	98.7 (0.1)	31.1 (5.8)	81.1 (4)	45 (6.1)	77.9 (1.8)	66.8 (27.8)
Regularized discriminant analysis	98.3 (0.2)	22.9 (11.3)	91.5 (3)	46.9 (6.3)	82.5 (2.8)	68.4 (32.2)
Mixture discriminant analysis^g^	98.4 (0.2)	40.6 (6.9)	87.5 (3.1)	42.6 (11.3)	78.9 (3.8)	69.6 (26.5)
Kernel PLS^h^ discriminant analysis	99.7 (0.1)	0 (0)	90.3 (1)	0 (0)	41.7 (3.1)	46.3 (47.7)
Bagged CART^i^	98.7 (0.2)	22.7 (7.8)	89.6 (2.6)	31.1 (8.8)	72.3 (4.6)	62.9 (34.3)
Quinlan’s C5.0 algorithm	98.8 (0.4)	13.3 (9.6)	91.6 (3.5)	29 (14.5)	72.4 (7.8)	61 (38.1)
K nearest neighbors	98.8 (0.2)	18.9 (7.7)	92 (2.4)	31.4 (9.9)	70.2 (4.3)	62.8 (35.8)
Support vector machine	98.9 (0.2)	1.1 (2.6)	95.6 (1.5)	23.3 (15.8)	76.2 (2.8)	59 (44.3)
Neural network	98.7 (0.3)	14.1 (10.7)	94 (2.4)	30.7 (15.6)	77.1 (5.5)	62.9 (38.3)

^a^NEG: uninfected-unvaccinated.

^b^VAC1: vaccinated with 1 dose.

^c^VAC2: vaccinated with 2 doses.

^d^INF.VAC: infected-vaccinated regardless of the number of doses.

^e^INF: infected-unvaccinated.

^f^Mean predictive performance across the 5 statuses.

^g^Selected model.

^h^PLS: partial least squares.

^i^CART: classification and regression tree.

### Prediction for the SERPICO Serosurvey

The model was applied to the French nationwide serosurveillance survey SERPICO (n=23,886). The consistency of the predictions was confirmed by comparing the predicted results to internal and external reference information (Multimedia Appendix 1). It follows that the estimation of the vaccinated proportion of the French population was correctly predicted, especially between March 2020 and June 2021. Predictions were less accurate for the last 2 periods as the proportion of individuals with a complete vaccination scheme (VAC2 and INF.VAC) tended to be underestimated in the prediction.

**Figure 4:**
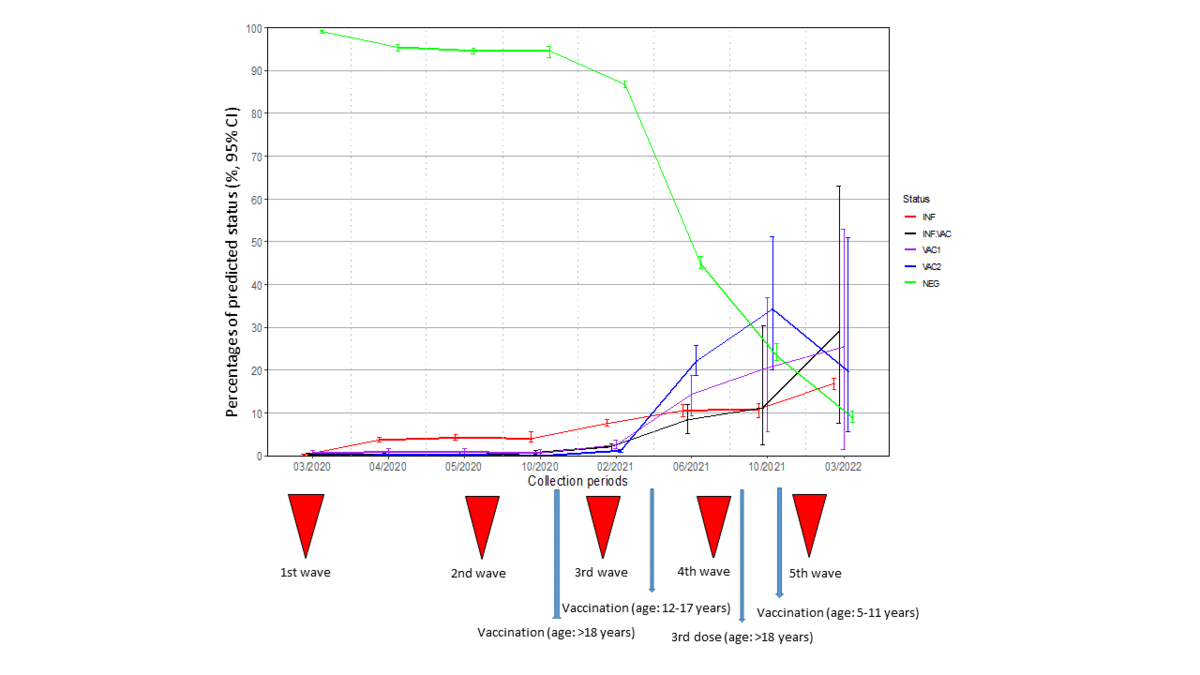
Percentages (95% CI) of SARS-CoV-2 predicted status for the 8 collection periods. INF: infected-unvaccinated; INF.VAC: infected-vaccinated regardless of the number of doses; NEG: uninfected-unvaccinated; VAC1: vaccinated with 1 dose; VAC2: vaccinated with 2 doses.

The average percentages of the 5 predicted SARS-CoV-2 immune statuses are shown in Figure 4 for the 8 sampling periods. For the first 4 periods, individuals were mostly predicted as NEG (March 2020: mean 99.1%, SD 0.2%; October 2020: mean 94.7%, SD 0.4%). Between February 2021 and March 2022, the number of INF individuals increased from 7.5% (SD 0.4%) to 16.8% (SD 0.7%). From June 2021, the numbers of vaccinated (VAC1 and VAC2) and INF.VAC individuals increased in relation with the rollouts of vaccination for the older population from the end of December 2020, for any person older than 12 years from June 2021, and for children aged 5 to 11 years from December 2021 in France. The prediction performances of the model were usually better for the most common statuses (NEG and INF). The INF.VAC status was better predicted in the 4 later periods during the vaccination rollout.

**Figure 5:**
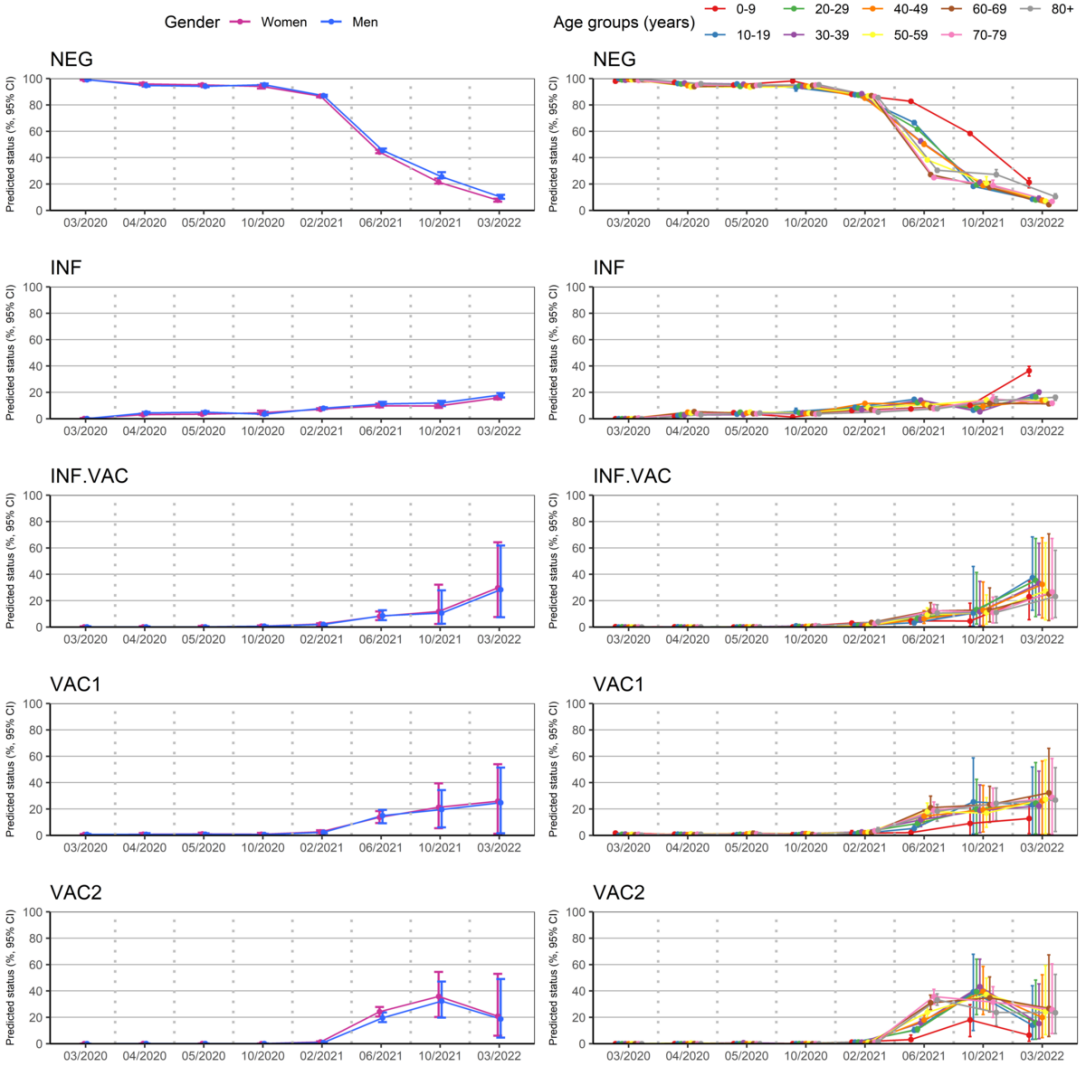
Percentages (95% CI) of SARS-CoV-2 predicted statuses for the 8 collection periods according to gender (left panel) and age group (right panel) in the SERPICO data set. INF: infected-unvaccinated; INF.VAC: infected-vaccinated regardless of the number of doses; NEG: uninfected-unvaccinated; VAC1: vaccinated with 1 dose; VAC2: vaccinated with 2 doses.

No difference in SARS-CoV-2 predicted status was observed between genders, except in October 2021 (Figure 5, left panel). At this time, the percentage of predicted NEG men was higher than that of women. No other significant difference was observed due to large CIs associated with the VAC1, VAC2, and INF.VAC predictions.

The percentage of INF individuals was similar in all age groups over the 7 sampling periods (Figure 5, right panel). In June 2021, the proportions of individuals predicted as VAC1 and VAC2 increased first in older age groups (60-69, 70-79, and older than 80 years) as vaccination was rolled out for these age groups first. Conversely, the proportion of individuals predicted to be NEG remained higher for the younger age groups during the same period. The proportion of NEG individuals was still higher in children aged 0 to 9 years in comparison with other age groups in October 2021 because vaccination was open only to children aged 5 to 11 years.

**Figure 6:**
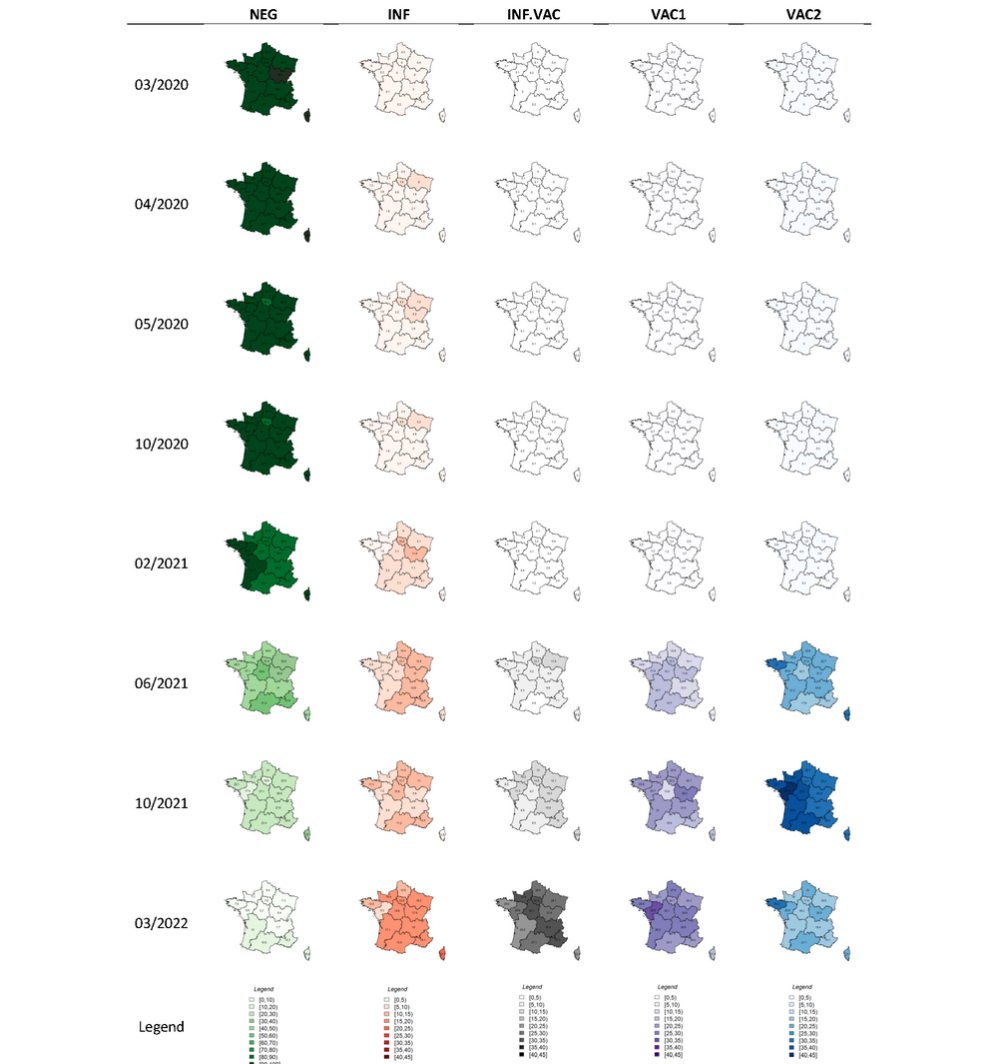
Percentages of SARS-CoV-2 predicted status for the 8 collection periods according to regions in France for the SERPICO data set. INF: infected-unvaccinated; INF.VAC: infected-vaccinated regardless of the number of doses; NEG: uninfected-unvaccinated; VAC1: vaccinated with 1 dose; VAC2: vaccinated with 2 doses. For a higher-resolution version of this figure, see Multimedia Appendix 2.

Predictions for the 5 SARS-CoV-2 statuses can be illustrated according to French administrative regions (Figure 6). The epidemic first progressed in the Eastern part of France between March and May 2021 and then spread to the Paris region and to the northern part of France in October 2021. Spatial variability was observed in immune status, with the predicted fraction of vaccinated individuals being higher in the western part of France than in other regions in June and October 2021.

## Discussion

### Serological Assays

The objective of our study was to predict the infected or vaccinated status of the individuals enrolled in the SARS-CoV-2 serosurveillance survey without prior information on their SARS-CoV-2 infection or vaccination status. For that purpose, reference data on serological profiles of seronegative, infected, and vaccinated individuals from another French cohort were used to build the predictive model. This approach was possible because serological testing was carried out using the same assays developed by the National Reference Centre for Respiratory Diseases of the Institut Pasteur, ensuring the comparability of the serological results between studies.

LuLISA-S is a sensitive assay for identifying individuals exposed to SARS-CoV-2 infection or vaccination. As the vaccines used in France target the SARS-CoV-2 spike protein only, we were confident in the ability of the LuLISA-N assay to distinguish naturally infected individuals from vaccinated ones in the CURIE-O-SA and SERPICO surveys. The LN and LS assays were studied in the context of infection and vaccination [13]. What was less expected was the determinant role of the PNT assay in differentiating INF.VAC individuals from others. The infected status was characterized by a higher pseudoneutralization capacity than that in vaccinated individuals only, confirming previous observations of the higher protection conferred by infection followed by vaccination [4]. Therefore, the 3 serological assays were complementary and essential to obtain a clear distinction between all SARS-CoV-2 statuses in the population, despite the fact that using 3 assays is costly and time-consuming for serosurveillance purposes.

### Machine Learning and Data-Driven Analysis

To build models predicting the SARS-CoV-2 status from serological assays, a machine learning procedure was applied. This procedure made it possible to compare a large number of classification models and select the most predictive one. This kind of procedure was previously used for prediction of SARS-CoV-2 status based on serological test results in a population vaccinated with whole virion vaccines [8].

The proposed procedure was based on raw serological results without setting any thresholds, contrary to previous works [3]. The thresholds used in the SERPICO survey were designed to maximize the specificity of the assays in a context of a low prevalence of SARS-CoV-2 infection in France from March 2020 to April 2021. In this study, adopting a data-driven strategy without any assay threshold enabled us to capture the evolution of the SARS-CoV-2 epidemic without modifying the assay interpretations over time [14]. The proposed model exhibited similar performance to the standard method for distinguishing negative versus positive SARS-CoV-2 immune statuses; however, it provided additional information about the positive status (ie, vaccinated, infected, or both; Multimedia Appendix 1).

To ease the use of the model, an R-shiny application was developed for the National Reference Centre and implemented in a user-friendly environment. Input data from the serological assays can be uploaded as a data frame and the most likely SARS-CoV-2 status is given [15].

### Vaccination Impact on Seroprevalence

Application of the predictive model to serosurveillance results gave useful insights for interpreting the evolution of SARS-CoV-2 seroprevalence in France. Between June and October 2020, the proportion of individuals who were seronegative to SARS-CoV-2 infection was still higher than 90% despite the first 2 SARS-CoV-2 waves that greatly impacted health services in March and September 2020. The observed quasistability of seroprevalence over this period could be explained by an increase in seroprevalence due to the waves of infections, tempered by the fairly rapid decrease in anti–SARS-CoV-2 antibodies.

Between October 2020 and March 2022, the proportion of seronegative individuals decreased. As the proposed predictions showed that the proportion of INF individuals did not increase over the same period, the decrease of the seronegative population could be mainly attributed to vaccination. Additionally, the decrease of the NEG population occurred earlier in the older age groups (older than 60 years) who were targeted first by the vaccination program. A part of this older population also benefited from a hybrid immunity due to vaccination and infection, although to a lesser degree than the rest of the adult population. On the contrary, two-thirds of children aged 0 to 9 years were still seronegative to SARS-CoV-2 in October 2021 due to a later rollout of vaccination for children aged 5 to 11 years. The expected percentages infected children aged 0 to9 years increased from 1.3% to 36.4% from October 2021 to March 2021. This clearly shows the impact of the Omicron strain on this poorly vaccinated population. All together, these results confirm that population immunity toward SARS-CoV-2 infection would progress very slowly without vaccination in the French population [16]. Moreover, such a strategy—necessarily combined with continued restrictive measures aimed to avoid overwhelming the health care system—would have had a tremendous impact on the economy and mental health.

### Consistency of the Predictions

The proposed predictive model produced SARS-CoV-2 immune status predictions in accordance with the observed SARS-CoV-2 epidemiological situation in France from March 2020 to March 2022 as results were only given in terms of infection prevalence (Multimedia Appendix 1). Predictions by region, gender, and age were consistent with the epidemiological weekly observations of the SARS-CoV-2 epidemic [17].

The consistency of the predictions was largely due to the use of 3 complementary serological assays that enabled us to finely distinguish the 5 SARS-CoV-2 statuses through a machine learning procedure. The main limit of the predictive models is associated with the data from the cohort used for calibration and validation of the models. Indeed, women and middle-aged individuals were overrepresented in the CURIE-O-SA cohort. The lack of profile diversity in the calibration and validation data set may explain why including the covariates (gender and age) did not improve model predictions. In addition, the CURIE-O-SA study took place from April 2020 to November 2021; therefore, it did not capture key evolutions in the SARS-CoV-2 epidemic in France, such as the emergence of the Omicron variant (November 2021) that provoked a different response to the PNT assay used in our study. In addition, infected and vaccinated individuals became more frequent in the overall population over time. This profile of individuals and individuals vaccinated with 1 dose were rare in the CURIE-O-SA cohort, leading to poor accuracy in the prediction of those statuses. The high percentages of individuals predicted to be vaccinated with 1 dose in October 2021 and March 2022 may have been due to the waning immunity of individuals vaccinated with 2 doses for a long time [5,18-20]. Lastly, the CURIE-O-SA cohort did not cover the rollout of the third vaccination dose. The validity limits of the predictive models were thus reached in October 2021, as exemplified by the overlapping CIs of the different percentages of vaccination statuses. The collection of new reference results including both results for the 3 serological assays and SARS-CoV-2 infection and vaccination history of the individuals is needed to update the model. Maintaining serosurveillance distinguishing vaccinated or infected populations is of interest to describe the evolution of SARS-CoV-2 immunity in the overall population and to understand immunity waning over time, but additional samples are needed for that purpose.

### Conclusion and Perspectives

A predictive model of individual vaccination or infection status with respect to SARS-CoV-2 was proposed based on 3 complementary serological assays and based on a machine learning procedure. This model was applied to the French nationwide serosurveillance survey from 2020 to 2022 to estimate the proportions of the French population that were seronegative, infected, vaccinated (1 or 2 doses), or infected and vaccinated, as this data set included no prior information on the SARS-CoV-2 infection or vaccination status of the individuals. This allowed us to follow the level of SARS-CoV-2 infection and the vaccine response profile of the French population over time.

Combining the results from the serosurveillance survey with previously acquired results from a cohort studied longitudinally improved the information retrieved from serosurveillance while keeping its protocol simple and easy to implement (no need to collect SARS-CoV-2 information on a large sample of individuals). We think that such a combination strategy is of interest to improve serosurveillance of emerging vaccine-preventable diseases. The results of our predictive model make it possible to measure the crucial contribution of SARS-CoV-2 vaccination to rapidly reach a level of collective immunity that has made it possible to relax sanitary measures without overloading the health care system. Indeed, population immunity toward SARS-CoV-2 infection would have progressed very slowly without vaccination.

## Appendix

Multimedia Appendix 1 Consistency of the SERPICO predictions.

Multimedia Appendix 2 Percentages of SARS-CoV-2 predicted status for the 8 collection periods according to regions in France for the SERPICO data set. INF: infected-unvaccinated; INF.VAC: infected-vaccinated regardless of the number of doses; NEG: uninfected-unvaccinated; VAC1: vaccinated with 1 dose; VAC2: vaccinated with 2 doses.

## Abbreviations

INF: infected-unvaccinated
INF.VAC: infected-vaccinated regardless of the number of doses
LN: nucleocapsid
LS: spike protein
LuLISA: luciferase-linked immunosorbent assay
NEG: uninfected-unvaccinated
PNT: pseudoneutralization assay
RT-PCR: reverse transcription polymerase chain reaction
VAC1: vaccinated with 1 dose
VAC2: vaccinated with 2 doses
