# Supplementary material for: Application of Machine Learning Prediction of Individual SARS-CoV-2 Vaccination and Infection Status to the French Serosurveillance Survey From March 2020 to 2022: Cross-Sectional Study
Source: JMIR Public Health Surveill. 2023 Nov 28;9:e46898. doi: 10.2196/46898 (PMC10716765; doi:10.2196/46898)
Supplement: Multimedia Appendix 1 [file publichealth_v9i1e46898_app1.docx]

**Supplementary Material 1: Consistency of the SERPICO predictions**

The predicted status issued from the application of the model to the SERPICO serological results were compared to internal and external data describing SARS-CoV-2 epidemiological situation in France to assess the consistency of the predictions. They were first compared to seroprevalence per period estimated from the standard method considered as a gold standard (i.e., POS = log10LN > 4.60 or log10LS > 4.44 or log10PNT < 4.54). For this purpose, subjects were considered as positive if they were predicted as VAC1, VAC2, INF.VAC or INF. The overall predictive ability of the model is 98.1% [97.8; 98.3] (99.0% for POS and 96.2% for NEG). Results are detailed in Supplementary Table 1a.

Then, the model predictions were compared to the known vaccination coverage of the French population (https://www.data.gouv.fr/fr/datasets/donnees-relatives-aux-personnes-vaccinees-contre-la-covid-19-1/#resources). For each period, the percentage of the population vaccinated with one dose or two doses at more than two weeks was extracted from the national database. As the full vaccination scheme includes both subjects vaccinated with two doses or more, and vaccinated with one dose and infected, this scheme was compared to the sum of the predicted VAC2 and INF.VAC subjects. To have a relevant comparison with the national base, predicted percentages were post-stratified by age, gender and region. Results are detailed in Supplementary Table 1b. The estimation of the vaccinated proportion of the French population was correctly predicted, especially for the first six periods (Supplementary Table 1b). Predictions were less accurate for the last two periods as the proportion of subjects with a complete vaccination scheme (VAC2 + INF.VAC) tended to be underestimated in the prediction (third dose, new variants). The underestimation was due to misclassification of VAC2 subjects into VAC1 subjects by the predictive model. It highlights that the vaccine coverage overestimated the SARS-CoV-2 immunity level as estimated from the three serological assays in the population.

**Supplementary Table 1a.** Expected (standard method) and predicted (mixture discriminant analysis) seroprevalences per collection period (SERPICO survey, N= 23,886 subject × date units, March 2020-March 2022, France).

| Periods | N | Expected seroprevalence | Predicted seroprevalence |
| --- | --- | --- | --- |
| 9-15/03/2020 | 3,221 | 0.7% | 0.8% |
| 6-12/04/2020 | 3,084 | 4.3% | 4.8% |
| 11-17/05/2020 | 2,879 | 5.0% | 5.6% |
| 5-11/10/2020 | 2,742 | 4.9% | 6.8% |
| 8-14/02/2021 | 2,979 | 12.9% | 13.8% |
| 7-13/06/2021 | 3,133 | 55.0% | 54.0% |
| 18-22/10/2021 | 2,913 | 76.7% | 77.4% |
| 14-20/03/2022 | 2,935 | 91.1% | 91.8% |

**Supplementary Table 1b.** Expected (nationwide vaccine coverage data) and post-stratified predicted (mixture discriminant analysis) percentages of subjects vaccinated with one dose and with a full vaccination scheme (=vaccinated with two doses + infected and vaccinated). (SERPICO survey, N= 23,886 subject × date units, March 2020-March 2022, France).

|  |  | Expected vaccine coverage | | | Predicted vaccine coverage | | |
| --- | --- | --- | --- | --- | --- | --- | --- |
| Periods | **N** | **Vaccinated with one dose** | **Full vaccination scheme** | **Vaccinated population** | **Post-stratified VAC1** | **Post-stratified VAC2+INF.VAC** | **Post-stratified VAC1+VAC2+INF.VAC** |
| 9-15/03/2020 | 3,221 | 0% | 0% | **0%** | 0.6% | 0.1% | **0.7%** |
| 6-12/04/2020 | 3,084 | 0% | 0% | **0%** | 0.8% | 0.2% | **1.0%** |
| 11-17/05/2020 | 2,879 | 0% | 0% | **0%** | 0.7% | 0.3% | **1.0%** |
| 5-11/10/2020 | 2,742 | 0% | 0% | **0%** | 0.4% | 0.9% | **1.3%** |
| 8-14/02/2021 | 2,979 | 2.1% | 0% | **2.1%** | 2.4% | 3.0% | **5.4%** |
| 7-13/06/2021 | 3,133 | 16.8% | 19.4% | **36.2%** | 11.6% | 29.0% | **40.6%** |
| 18-22/10/2021 | 2,913 | 2.0% | 66.0% | **68.0%** | 27.5% | 38.2% | **65.7%** |
| 14-20/03/2022 | 2,935 | 1.0% | 70.4% | **71.4%** | 34.4% | 39.8% | **74.2%** |
