# Supplementary figures and images for: Application of Machine Learning Prediction of Individual SARS-CoV-2 Vaccination and Infection Status to the French Serosurveillance Survey From March 2020 to 2022: Cross-Sectional Study
Source: JMIR Public Health Surveill. 2023 Nov 28;9:e46898. doi: 10.2196/46898 (PMC10716765; doi:10.2196/46898)

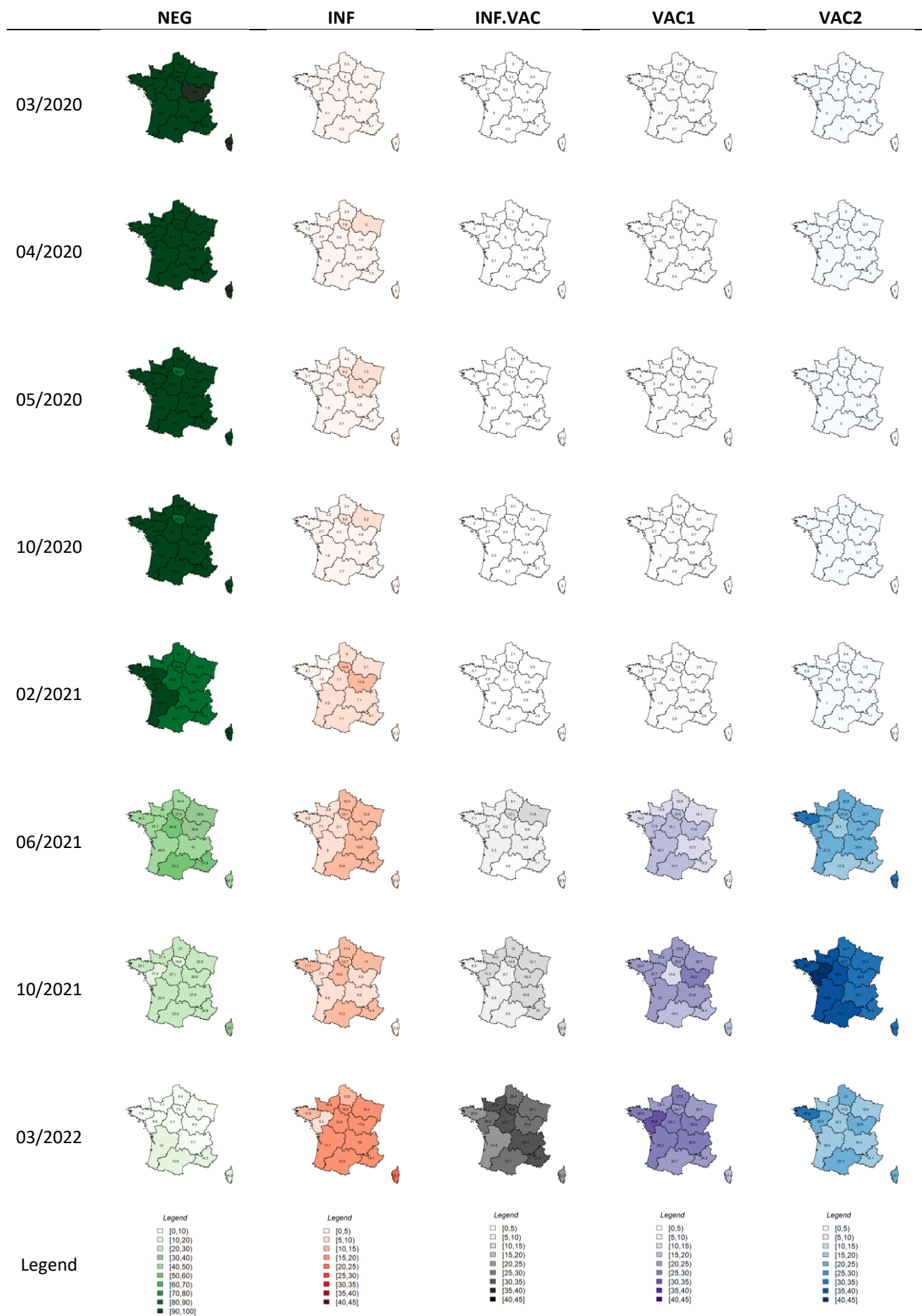

Supplement: Multimedia Appendix 2 [file publichealth_v9i1e46898_app2.pdf]
